# Cryptanalysis and Improvement of a Privacy-Preserving Three-Factor Authentication Protocol for Wireless Sensor Networks

**DOI:** 10.3390/s19214625

**Published:** 2019-10-24

**Authors:** Km Renuka, Sachin Kumar, Saru Kumari, Chien-Ming Chen

**Affiliations:** 1 Department of Mathematics, Ch. Charan Singh University, Meerut, Uttar Pradesh 250004, India; baliyanrenuka@gmail.com (K.R.); saryusiirohi@gmail.com (S.K.); 2 Department of Computer Science and Engineering, Ajay Kumar Garg Engineering College, Ghaziabad 201009, India; imsachingupta@rediffmail.com; 3 College of Computer Science and Engineering, Shandong University of Science and Technology, Qingdao 266590, China

**Keywords:** wireless sensor networks, multi-factor authentication, fuzzy extractor, anonymity, provably security

## Abstract

Wireless sensor networks (WSNs) are of prominent use in unmanned surveillance applications. This peculiar trait of WSNs is actually the underlying technology of various applications of the Internet of Things (IoT) such as smart homes, smart cities, smart shopping complexes, smart traffic, smart health, and much more. Over time, WSNs have evolved as a strong base for laying the foundations of IoT infrastructure. In order to address the scenario in which a user wants to access the real-time data directly from the sensor node in wireless sensor networks (WSNs), Das recently proposed an anonymity-preserving three-factor authentication protocol. Das’s protocol is suitable for resource-constrained sensor nodes because it only uses lightweight cryptographic primitives such as hash functions and symmetric encryption schemes as building blocks. Das’s protocol is claimed to be secure against different known attacks by providing formal security proof and security verification using the Automated Validation of Internet Security Protocols and Applications tool. However, we find that Das’s protocol has the following security loopholes: (1) By using a captured sensor node, an adversary can impersonate a legal user to the gateway node, impersonate other sensor nodes to deceive the user, and the adversary can also decrypt all the cipher-texts of the user; (2) the gateway node has a heavy computational cost due to user anonymity and thus the protocol is vulnerable to denial of service (DoS) attacks. We overcome the shortcomings of Das’s protocol and propose an improved protocol. We also prove the security of the proposed protocol in the random oracle model. Compared with the other related protocols, the improved protocol enjoys better functionality without much enhancement in the computation and communication costs. Consequently, it is more suitable for applications in WSNs

## 1. Introduction

Wireless sensor networks (WSNs) play a pivotal role in the origin and propagation of the IoT, the notion that each object (virtual or physical) can be sensed, identified, accessed and interconnected via the Internet within a dynamic ubiquitous network. Wireless sensor networks (WSNs) are networks composed of a large number of randomly distributed sensor nodes. These sensor nodes jointly perceive environmental information and transmit the perceived information to the gateway node through a self-organizing multi-hop network. WSNs are widely used in battlefield situational awareness, environmental monitoring, medical care and water quality monitoring due to their characteristics of self-organization and reliability. Sensor nodes are usually deployed in unmanned or hostile areas, and their perceived information is of high value, so users must pass identity authentication before obtaining the information perceived by the sensor nodes [1,2].

Usually, the gateway node stores the information transmitted by the sensor node, and the user sends the request for data to the gateway node and obtains the data stored by it. However, in many scenarios with high demand for real-time data application, such as battlefield situational awareness and enemy detection, users need to obtain real-time data directly from sensor nodes. Various authentication protocols have been proposed for wireless sensor networks scenario, such as in [2,3,4,5,6,7,8,9,10,11,12,13,14,15,16,17,18,19,20]. In order to describe the security authentication requirements in this scenario, Das designed a two-factor authentication protocol using smart cards and passwords [3]. Two-factor protocol combines two different authentication methods. An attacker can only destroy the security of the protocol by corrupting all authentication factors and corrupting only one authentication factor will not affect the security of the protocol. Das claims that their protocol can resist known attacks such as replay attacks, password guessing attacks, and impersonation attacks. However, Nyang et al. found that the Das’s protocol could not resist offline dictionary attack, node capture attack, and the protocol could not protect the response information of the query [4]. Nyang et al. proposed an improved protocol to overcome the shortage of Das’s protocol. Chen et al. pointed out that the Das protocol failed to realize two-way authentication and made corresponding improvements [5]. In addition, He et al. found that Das’s protocol could not resist insider attacks and internal malicious user impersonation attacks [6]. Later, Khan et al. pointed out that in Das’s protocol, attackers can bypass the authentication of gateway node and can directly obtain information from sensor node. Khan et al. also identified that this protocol cannot resist privileged insider attack, does not provide any password update mechanism, and fails to realize the two-way authentication between gateway node and sensor node [7]. Khan et al. proposed an improved protocol to overcome the security flaws in the Das protocol. However, Sun et al. pointed out that Khan et al.’s improved protocol was still subject to gateway node impersonation attack and privileged insider attack, and attackers could still bypass the authentication of gateway node and directly obtain information from sensor node [8]. Aiming at the security vulnerability of Khan et al.’s protocol, Sun et al. proposed a two-factor authentication protocol and proved the security of their protocol under BR model [9]. Yuan et al. also found that Khan et al.’s protocol could not provide non-repudiation, could not resist smart card theft attacks, and could not achieve two-way authentication between users and sensor nodes [10]. Yuan et al. then designed a multi-factor authentication protocol using biological authentication and proved the security of the protocol by using GNY logic [11]. Wu et al. [12] designed a provably secure three-factor user authentication protocol for wireless sensor networks. Their scheme attains a number of desirable features but the computational and communication overheads are high.

Recently, Das proposed a multi-factor authentication protocol combining password, smart card and biological information [18]. Their protocol only adopts lightweight cryptographic components, such as hash function and symmetric encryption algorithm, so it confirms to the characteristics of limited resource of sensor nodes in wireless sensor network. In addition, Das uses formalized proof method and an automatic protocol verification tool to prove the security of its protocol. However, we found that Das’s protocol has the following security vulnerabilities: (1) the attacker can impersonate the user to the gateway node by using the captured sensor node, and can decrypt all the encrypted data of the user; (2) anonymity requires a lot of computing by the gateway node, which will lead to denial of service attack on the gateway node. Therefore, the protocol does not have user anonymity. In view of the above security vulnerabilities, we further improve the Das’s protocol and give the formalized security proof of the improved protocol under the random prediction model. It can be seen from the efficiency analysis that compared with similar protocols, our improved protocol has higher security while having considerable computing and communication costs, so it is more compatible with the application requirements of WSN.

The remaining sections of this article are arranged as follows: the second section summarizes the symbols used in this paper and reviews the Das’s protocol; Section 3 shows our attack on Das’s protocol. Section 4 describes our improved protocol; Section 5 gives the formal security proof of the improved protocol. The computational efficiency and communication efficiency of the improved protocol are compared in Section 6. Finally, the article is summarized in Section 7.

## 2. Review of Das’s Protocol

In this section, we first summarize the symbols used in the paper and their corresponding meanings in Table 1, and then briefly review the multi-factor authentication protocol with anonymity designed by Das [18]. Das designed a multi-factor authentication protocol including registration phase, login phase, authentication and key establishment phase, password & biological information update phase, and sensor node dynamic join phase. We focus on the first three core phases of the protocol. For more details of the protocol, readers may refer to [18].

### 2.1. Registration Phase

During the registration phase, a legitimate user $U_{i}$ registers with the gateway node *GW* over a secure channel. The registration phase includes the following steps:

StepR1: The user $U_{i}$ selects his or her identity $ID_{i}$, password $PW_{i}$ and samples its biometric template $B_{i}$, and then randomly generates a 1024-bit key *K*. The user $U_{i}$ computes user $Gen(B_{i})=(\sigma_{i},\tau_{i})$ through the biometric key generation algorithm Gen(•) in the fuzzy extractor [21], where is the bio-key, and $\tau_{i}$ is the public information for recovering $\sigma_{i}$.

StepR2: The user $U_{i}$ calculates the masked password information $RPW_{i}=h(ID_{i}||K||PW_{i})$ and sends the message $\left(ID_{i},RPW_{i}\right)$ to the gateway node *GW* over the secure channel.

StepR3: After receiving the registration request, the gateway node *GW* generates a 1024-bit key $X_{S}$ and calculates $r_{i}=h(ID_{i}||X_{S})$; the gateway node *GW* issues a smart card $SC_{i}$ to the user $U_{i}$ through the secure channel, where the smart card contains information $\{r_{i},h(•)\}.$

StepsR4: After receiving the smart card $SC_{i}$, the user $U_{i}$ calculates $e_{i}=h(ID_{i}||\sigma_{i})⊕K$, $f_{i}=h(ID_{i}||RPW_{i}||\sigma_{i})$, and $r_{i}^{*}=r_{i}⊕h(ID_{i}||K)$,. Finally, the user $U_{i}$ replaces $r_{i}^{*}$ with $r_{i}$ and save the information and $\left\{e_{i},f_{i},\tau_{i},Gen(•),Rep(•)\right\}$ to the smart card.

### 2.2. Login Phase

If a user $U_{i}$ wants to pass the authentication of the gateway node *GW* and obtain real-time information from the sensor node, the user needs to perform the following steps:

StepL1: The user $U_{i}$ first inserts his smart card $SC_{i}$ into the card reader, then enters his or her identity $ID_{i}$, password $PW_{i}$, and samples its biometric template $B_{i}^{*}$.

StepL2: The smart card $SC_{i}$ uses the fuzzy key extractor’s bio-key recovery algorithm to calculate, $\sigma_{i}^{*}=Rep(B_{i}^{*},\tau_{i})$, $K^{*}=h(ID_{i}||\sigma_{i}^{*})⊕e_{i}$, $RPW_{i}^{*}=h(ID_{i}||K^{*}||PW_{i})$ and $f_{i}^{*}=h(ID_{i}||RPW_{i}^{*}||\sigma_{i}^{*}),$ the smart card $SC_{i}$ verifies whether $f_{i}^{*}$ is equal to the stored $f_{i}$. If they are equal, the smart card $SC_{i}$ passes the verification of the user’s password and biometric information; otherwise the smart card refuses to run rest of the protocol.

StepL3: The smart card $SC_{i}$ calculates $M_{1}=r_{i}^{*}⊕h(ID_{i}||K^{*})$ and generates a random number $RN_{U_{i}}$. Suppose the user $U_{i}$ wants to get the information collected by the sensor node $SN_{j}$, then the smart card $SC_{i}$ calculates $M_{2}=M_{1}⊕RN_{U_{i}}$ and $M_{3}=h(ID_{i}||ID_{SN_{j}}||M_{1}||RN_{U_{i}}||T_{1})$, where $T_{1}$ is the current timestamp of the system. The smart card $SC_{i}$ finally sends a message $\left(ID_{SN_{j}},M_{2},M_{3},T_{1}\right)$, to the gateway node *GW*.

### 2.3. Authentication and Key Establishment Phase

After receiving the authentication request information $\left(ID_{SN_{j}},M_{2},M_{3},T_{1}\right)$ of the user, the gateway node *GW* performs the following steps:

StepA1: The gateway node *GW* first verifies the validity of the timestamp $T_{1}$, that is, suppose the message is received at the time $T_{2}$, and verifies whether $|T_{2}-T_{1}|\leq \Delta T$ is valid, where ΔT is the maximum delay allowed for the message transmission in the sensor network. If the above verification is passed, the gateway node *GW* further calculate $M_{4}=h(ID_{i}||X_{S})$, $M_{5}=M_{2}⊕M_{4}=RN_{U_{i}}$, $M_{6}=h(ID_{i}||ID_{SN_{j}}||M_{4}||M_{5}||T_{1})$.The gateway node *GW* verifies whether $M_{6}=M_{3}$ is valid. If so, the user identity is legal; otherwise, the gateway node *GW* terminates the protocol.

StepA2: The gateway node *GW* calculates the encrypted cipher-text $M_{7}=E_{MK_{SN_{j}}}(ID_{i},ID_{SN_{j}},M_{5},h(M_{4}),T_{1},T_{3})$, where $MK_{SN_{j}}$ is the master key shared by the gateway node *GW* and the sensor node $SN_{j}$ and $T_{3}$ is the current timestamp of the system. The last gateway node *GW* sends a message $\left(ID_{SN_{j}},M_{7}\right)$ to the sensor node $SN_{j}$.

StepA3: When the sensor node $SN_{j}$ receives the message $\left(ID_{SN_{j}},M_{7}\right)$ at the time $T_{4}$, it first decrypts the message $M_{7}$ with its master key $MK_{SN_{j}}$, and then verifies whether the decrypted identity information $ID_{SN_{j}}$ is correct and further verifies whether $|T_{4}-T_{3}|\leq \Delta T$ is established. If it is established, the message is legal, otherwise the sensor node $SN_{j}$ terminates the protocol operation.

StepA4: When the sensor node $SN_{j}$ generates a random number $RN_{SN_{j}}$, calculates the session key $SK_{ij}=h(ID_{i}||ID_{SN_{j}}||h(M_{4})||M_{5}||RN_{SN_{j}}||T_{1}||T_{5})$ shared with the user, where $T_{5}$ is the current timestamp of the system; in addition, the sensor node $SN_{j}$ calculates $M_{8}=h(SK_{ij})$ and $M_{9}=M_{5}⊕RN_{SN_{j}}⊕ID_{i}$ finally $SN_{j}$ sends $\left(M_{8},M_{9},T_{5}\right)$ to the user $U_{i}$.

StepA5: When the user $U_{i}$ receives the message $\left(M_{8},M_{9},T_{5}\right)$ at the time $T_{6}$, first verifies whether

$|T_{6}-T_{5}|\leq \Delta T$ is true. If true, user $U_{i}$ calculate $M_{10}=M_{9}⊕RN_{U_{i}}⊕ID_{i}=RN_{SN_{j}}$ $SK_{ij}^{’}=h(ID_{i}||ID_{SN_{j}}||h(M_{1})||RN_{U_{i}}||M_{10}||T_{1}||T_{5})$, $M_{11}=h(SK_{ij}^{’})$, through their smart cards $SC_{i}$, as well. 

Finally, the user $U_{i}$ verifies whether $M_{11}=M_{8}$ is true. If true, the user $U_{i}$ accepts the protocol operation and uses the session key $SK_{ij}^{’}$ and the sensor node $SN_{j}$ to perform confidential data transmission in subsequent communication.

## 3. Security Analysis of Das’s Protocol

In this section we present a security analysis of the Das’s protocol. We found that the Das’s protocol has serious security vulnerabilities and security cannot be guaranteed.

### 3.1. Node Capture Attack

Since sensor nodes are typically deployed in unmanned or hostile areas, it is easy for an attacker to capture sensor nodes. It is usually required that sensor nodes are captured without affecting the remaining nodes and users in the network. However, in the Das’s protocol, if an attacker captures a sensor node $SN_{j}$, the master key $MK_{SN_{j}}$ of the node can be obtained, and then the attacker can perform the following two types of attacks.

#### 3.1.1. User Phishing Attack

After an attacker captures a sensor node $SN_{j}$, any user $U_{i}$ can send a data request to the node $SN_{j}$ through the gateway node *GW*, and the attacker can obtain the private key of user $U_{i}$ through the authenticated message and can spoof the user to the gateway node *GW*. After obtaining the message $\left(ID_{SN_{j}},M_{7}\right)$ sent by the gateway node *GW*, the attacker decrypts $M_{7}$ with master key $MK_{SN_{j}}$ and obtains $ID_{i},ID_{SN_{j}},M_{5},h(M_{4}),T_{1},T_{3}$. Note that $M_{5}=RN_{U_{i}}$ is a random number selected by the user $U_{i}$ and authentication request message $ID_{SN_{j}},M_{2},M_{3},T_{1}$.can be found by the attacker according to the timestamp $T_{1}$. The value $h(ID_{i}||X_{S})$ can be recovered using $M_{2}=h(ID_{i}||X_{S})⊕RN_{U_{i}}$. Where, $h(ID_{i}||X_{S})$ is the secret value which is used by user $U_{i}$ to prove identity to the server. An attacker can obtain data of all nodes of the whole sensor network by imitating users. Specifically, an attacker only needs to select a random number $RN_{U_{i}^{*}}$, then calculate $M_{2}^{*}=h(ID_{i}||X_{S})⊕RN_{U_{i}^{*}}$ and $M_{3}^{*}=h(ID_{i}||ID_{SN_{j}}||h(ID_{U_{i}}||X_{S})||RN_{U_{i}^{*}}||T_{1}^{*})$, where $T_{1}^{*}$ is the current timestamp of the system. Finally, the attacker sends a message $\left(ID_{SN_{k}},M_{2}^{*},M_{3}^{*},T_{1}^{*}\right)$ to the gateway node *GW*, where,$ID_{SN_{k}}$ is any sensor node that the attacker wants to get information. Obviously, the message $\left(ID_{SN_{k}},M_{2}^{*},M_{3}^{*},T_{1}^{*}\right)$ will be validated by the gateway node *GW*. Through the above attack, the attacker can obtain the data of all nodes in the whole network by imitating the user after capturing a sensor node.

#### 3.1.2. Sensor Node Phishing Attack

Similar to the above attack, the attacker can capture the sensor node $SN_{j}$ and can imitate the remaining sensor nodes to send false information to trick the user $U_{i}$. When the attacker intercepts the message sent by the user $U_{i}$, it only needs to use $h(ID_{U_{i}}||X_{S})⊕M_{2}$ to recover the random number selected by the user $U_{i}$, and then impersonate the sensor node $ID_{SN_{k}}$ to select the random number and return the message according to the description of the protocol. The attacker knows the user’s secret information $h(ID_{U_{i}}||X_{S})$, so the attacker can successfully imitate the remaining sensor nodes to trick the user $U_{i}$. Since sensor networks often involve sensitive military applications, false information can be sent to users through the above attacks, so node counterfeiting attacks can bring huge losses to the users.

It can be seen from the above two attacks that after the attacker captures a sensor node, not only the user’s secret information can be obtained, but the other sensor nodes of the network can be misused to send false information to the user, which brings huge security threat to the protocol.

### 3.2. Denial of Service Attack

Das’s protocol claims to implement anonymous protection for users, so the authentication information $\left(ID_{SN_{j}},M_{2},M_{3},T_{1}\right)$ of the user $U_{i}$ is not included in the user’s authentication information. When receiving a message, the gateway node *GW* needs to verify the validity of the authentication information without knowing the identity of the user. The protocol description does not explain how the gateway node *GW* knows the identity of the user. Therefore, according to the implementation of the protocol, only the exhaustive method can be used to verify the user’s authentication information, that is, for each possible identity ID, the gateway node *GW* calculates $M_{4}=h(ID_{i}||X_{S})$, $M_{5}=M_{2}⊕M_{4}$, $M_{6}=h(ID_{i}||ID_{SN_{j}}||M_{4}||M_{5}||T_{1})$ and further verifies whether $M_{6}=M_{3}$ is true. The above process will consume a large amount of computing resources from the gateway node *GW*. If the attacker impersonates the user to send an authentication request, the gateway node *GW* will not discover that the authentication request is invalid until traverse all registered users. Therefore, Das’s protocol cannot resist denial of service attacks due to user anonymity. Hence, Das’ protocol does not offer user anonymity due to errors in protocol design.

## 4. Improved Protocol

In view of the security vulnerabilities of Das’s protocol, we find that the root cause of node capture attack is that the user’s secret information is exposed to the sensor node incorrectly in the protocol design. In fact, in the authentication process, the sensor node and the gateway node authenticate through the shared key, and the sensor node should not get any secret information of the user. The essential reason is that the protocol does not realize user anonymity, which is a protocol design error. Based on the above analysis, this section presents our improved protocol.

### 4.1. Registration Phase

In the registration phase, a legitimate user $U_{i}$ registers with the gateway node *GW* through a secure channel. The registration stage includes the following steps:

StepR1: Users $U_{i}$ select their identity $ID_{i}$, password $PW_{i}$ and sample their bio-template $B_{i}$, and then calculate $Gen(B_{i})=(\sigma_{i},\tau_{i})$ using the bio-key generation algorithm Gen(•) in the fuzzy extractor, where $\sigma_{i}$ is the bio-key, and $\tau_{i}$ the public information for recovering $\sigma_{i}$.

StepR2: The user $U_{i}$ calculates the secret value $RPW_{i}=h(ID_{i}||\sigma_{i}||PW_{i})$ and sends the message $\left(ID_{i},RPW_{i}\right)$ to the gateway node *GW* through the secure channel.

StepR3: After receiving the registration request, the gateway node *GW* generates a 1024-bit key $X_{S}$ and chooses a random identity $DID_{i}$ for the user, then calculates $r_{i}=h(ID_{i}||DID_{i}||X_{S})$ the gateway node *GW* issues a smart card $SC_{i}$ containing information $\left\{r_{i}^{*}=r_{i}⊕RPW_{i},DID_{i},h(•)\right\}$ to the user $U_{i}$ through the secure channel. *GW* adds record $\left(DID_{i},ID_{i}\right)$ to its database and protects the database with its master key $X_{S}$.

StepR4: After receiving the smart card $SC_{i}$, the user $U_{i}$ deposits the information $\left\{\tau_{i},Gen(•),Rep(•)\right\}$ into the smart card.

### 4.2. Login Phase

If a user $U_{i}$ wants to authenticate the gateway node *GW* and obtain real-time information from the sensor node $SN_{j}$, the user needs to perform the following steps:

StepL1: The user $U_{i}$ first inserts his smart card $SC_{i}$ into the reader, then enters his identity $ID_{i}$, password $PW_{i}$ and sampled his biological template $B_{i}^{*}$.

StepsL2: Smart cards $SC_{i}$ computes $\sigma_{i}^{*}=Rep(B_{i}^{*},\tau_{i})$ using the bio-key recovery algorithm of the fuzzy extractor; then smart cards $SC_{i}$ computes,$RPW_{i}^{*}=h(ID_{i}||\sigma_{i}^{*}||PW_{i})$ and $M_{1}=r_{i}^{*}⊕RPW_{i}^{*}$.

StepL3: The smart card $SC_{i}$ calculates $K_{1}=h(M_{1},T_{1})$ and generates a random number $RN_{U_{i}}$, where,$T_{1}$ is the current timestamp of the system. If the user $U_{i}$ wants to get the information $SN_{j}$ collected by the sensor node $SN_{j}$, the smart card $SC_{i}$ calculates the ciphertext $C_{1}=E_{K_{1}}(DID_{i},RN_{U_{i}},T_{1})$. The smart card $SC_{i}$ finally sends a message $\left(DID_{i},ID_{SN_{j}},C_{1},T_{1}\right)$ to the gateway node *GW*.

### 4.3. Authentication and Key Establishment Phase

After receiving the authentication request information $\left(ID_{SN_{j}},M_{2},M_{3},T_{1}\right)$ of the user, the gateway node *GW* performs the following steps:

StepA1: Gateway node *GW* first verifies the validity of the timestamp $T_{1}$, that is, assuming that the message is received at time $T_{2}$, verifies whether $|T_{2}-T_{1}|\leq \Delta T$ is valid, where, ΔT is the maximum allowable delay of message transmission in sensor network. If the above authentication passes, the gateway node *GW* further searches for the corresponding user’s real identity $ID_{i}$ according to $DID_{i}$. If the database contains the above records, the gateway node *GW* calculates $K_{1}^{*}=h(h(ID_{i}||DID_{i}||X_{S}),T_{1})$ and decrypts the cipher-text $C_{1}$ using the temporary key. If the decrypted message contains the correct $DID_{i}$ and $T_{1}$, the user’s identity is legitimate; otherwise, the gateway node *GW* terminates the operation of the protocol.

StepA2: The gateway node *GW* calculates the encrypted cipher text $C_{2}=E_{MK_{SN_{j}}}(ID_{i},ID_{SN_{j}},RN_{U_{i}},T_{1},T_{3})$, where, $MK_{SN_{j}}$ is the master key shared by the gateway node *GW* and the sensor node $SN_{j}$, $RN_{U_{i}}$ is the random number obtained by decryption, and $T_{3}$ is the current timestamp of the system. Finally, the gateway node *GW* sends a message $\left(ID_{SN_{j}},C_{2}\right)$ to the sensor node $SN_{j}$.

StepA3: When the sensor node $SN_{j}$ receives the message $\left(ID_{SN_{j}},C_{2}\right)$ at the time $T_{4}$, it first decrypts the message $C_{2}$ with its master key $MK_{SN_{j}}$, then verifies whether the decrypted identity $ID_{SN_{j}}$ information is correct and further verifies whether $|T_{4}-T_{3}|\leq \Delta T$ is valid. If it is true, the message is legitimate; otherwise the sensor node $SN_{j}$ terminates the protocol.

StepA4: When the sensor node $SN_{j}$ generates a random number $RN_{SN_{j}}$ calculates the session key $SK_{ij}=h(ID_{i}||ID_{SN_{j}}||RN_{U_{i}}||RN_{SN_{j}}||T_{1}||T_{5})$, where $T_{5}$ is the current timestamp of the system; in addition, the sensor node $SN_{j}$ calculates $K_{2}=h(ID_{i},ID_{SN_{j}},RN_{U_{i}})$, $C_{3}=E_{K_{2}}(ID_{i},ID_{SN_{j}},RN_{SN_{j}})$, $Auth_{1}=h(SK_{ij}||RN_{U_{i}}||RN_{SN_{j}})$. Finally $SN_{j}$ sent $\left(Auth_{1},C_{3},T_{5}\right)$ to the user $U_{i}$.

StepA5: When the user $U_{i}$ receives the message $\left(Auth_{1},C_{3},T_{5}\right)$ at the time $T_{6}$, first verifies whether $|T_{6}-T_{5}|\leq \Delta T$ is valid. If true, the user $U_{i}$ calculates the temporary key $K_{2}=h(ID_{i},ID_{SN_{j}},RN_{U_{i}})$, and decrypts the cipher-text $C_{3}$. If the decrypted message contains the correct $ID_{i}$, $ID_{SN_{j}}$ then the user $U_{i}$ calculates $SK_{ij}^{*}=h(ID_{i}||ID_{SN_{j}}||RN_{U_{i}}||RN_{SN_{j}}||T_{1}||T_{5})$, where, $RN_{SN_{j}}$ is the random number which is decrypted from $C_{3}$. Finally, the user $U_{i}$ verifies the validity of the protocol. If it is validated, the user accepts the protocol to run and in the subsequent communication the session key $SK_{ij}^{*}$ is used to transmit confidential data with the sensor node $SN_{j}$.

### 4.4. Password and Biological Template Update Phase

Assuming the user wants to update the current password, he performs the following steps:

Step U1: The user $U_{i}$ inserts his/her smart card $SC_{i}$ into the card reader, then enters his/her identity $ID_{i}$, original password $PW_{i}$, new password $PW_{i}^{*}$, and samples his/her biometric template $B_{i}^{*}$.

Steps U2: The smart card $SC_{i}$ uses the biological key recovery algorithm Rep(•) of fuzzy extractor to calculate $\sigma_{i}^{*}=Rep(B_{i}^{*},\tau_{i})$. The smart card $SC_{i}$ then calculates $RPW_{i}=h(ID_{i}||\sigma_{i}^{*}||PW_{i})$ and $RPW_{i}^{*}=h(ID_{i}||\sigma_{i}^{*}||PW_{i}^{*})$. Finally, calculates $r_{i}^{*}⊕RPW_{i}⊕RPW_{i}^{*}$ and replaces the original $r_{i}^{*}$ with that value.

Similarly, the user $U_{i}$ can take similar steps to update the biological template.

## 5. Security Certificate

In this section, we formalize the security proof of our improved protocol. Firstly, we briefly review the two-factor protocol security model in [8] and extend it to the application environment of multi-factor protocol. Then we prove the security of our improved protocol under the extended model.

### 5.1. Formal Security Analysis of the Improved Protocol Using Random Oracle Model

The participants of the protocol include user U, gateway node *GW* and sensor node *SN*. To be simple, it is usually assumed that the gateway node *GW* is unique in the wireless sensor network. Each user can activate and run multiple session instances simultaneously. We use key $\Pi_{P}^{x}$ to represent the *x* th session instance of the protocol participant *P*, which can be a user, gateway node, or sensor node. Since the session key is shared by the user and sensor nodes, the session id $sid_{P}^{x}$ defining the user instance or sensor node instance $\Pi_{P}^{x}$ is a cascade of all messages (except the last one) that the instance sends and receives during the execution of the protocol. The partner id $pid_{P}^{x}$ defining the user instance or sensor node instance $\Pi_{P}^{x}$ is identified as the intended communicator with which the instance $\Pi_{P}^{x}$ wants to establish the session key.

Each user $U_{i}$ has three kinds of secret information, password $PW_{i}$, smart card $SC_{i}$, and biological template $B_{i}$, after the registration stage. Among them $PW_{i}$ is the low-entropy password, randomly selected from the password dictionary space. The gateway node *GW* has a long-term key $X_{S}$ and holds a list of records about user authentication information, where each record corresponds to a user; in addition, *GW* shares a high-entropy symmetric key $MK_{SN_{j}}$ with each sensor node $SN_{j}$. Each sensor node $SN_{j}$ stores a symmetric key $MK_{SN_{j}}$ shared with the gateway node. It is usually assumed that the sensor node is easily captured by the attacker, and the attacker can recover its master key $MK_{SN_{j}}$.

We call a user instance $\Pi_{U}^{x}$ and a sensor node instance $\Pi_{SN}^{y}$ partners if (1) both instances accept the protocol and generate a shared session key; (2) $sid_{U}^{x}=sid_{SN}^{y}$,(3) $pid_{U}^{x}=SN$ and $pid_{SN}^{y}=U$.

*A* as the attacker of the protocol, is a probabilistic polynomial time attacker and controls the communication network of the whole protocol. That is, the attacker can intercept, eavesdrop, delete, delay, modify and forge messages. In addition, according to the security definition of multifactor protocol, an attacker *A* can capture arbitrary sensor nodes and recover their stored keys, and can also arbitrarily obtain two types of authentication factors of user’s three types of authentication factors. It should be noted that attacker *A* are not allowed to corrupt gateway nodes, because once the gateway nodes are corrupted, any such protocol cannot guarantee session key security. We describe *A*’s ability by following instructions and inquiries:

*Execute*$\left(\Pi_{U_{i}}^{x},\Pi_{GW}^{y},\Pi_{SN_{j}}^{z}\right)$: This instruction describes *A*’s ability to passively eavesdrop conversational messages in network. Through this attack, *A* can obtain all publicly transmitted messages during the instance $\Pi_{U_{i}}^{x},\Pi_{GW}^{y},\Pi_{SN_{j}}^{z}$ running protocol.

*Send*$\left(\Pi_{P}^{x},m\right)$: This instruction describes *A*’s ability to attack instance $\Pi_{P}^{x}$ actively. Attacker *A* impersonates a protocol participant to send a message *m* to an instance $\Pi_{P}^{x}$ and gets the message returned by the instance $\Pi_{P}^{x}$ after receiving the message *m* according to the protocol description.

*Reveal*$\left(\Pi_{P}^{x}\right)$: This instruction can only be used for user instances or sensor node instances to characterize known key attacks. With this query, the attacker *A* will get the session key generated by the instance $\Pi_{P}^{x}$; if the instance $\Pi_{P}^{x}$ does not generate the session key, the query will be returned to denote invalidity.

*Corrupt* ($SN_{j}$): This instruction simulates capture attacks on sensor nodes. The attacker *A* will get the private key $MK_{SN_{j}}$ of the sensor node $SN_{j}$ and control the sensor node completely. If the attacker inquiries, the sensor node is said to be completely corrupted.

*Corrupt* ($U_{i}$): There are three types of corrupt inquiries about user $U_{i}$:

*Corrupt* ($U_{i},1$): The attacker *A* will get the password of user $U_{i}$ through this corrupt inquiry.

*Corrupt* ($U_{i},2$): The attacker *A* will get an effective biological template for the user through this corrupt inquiry.

*Corrupt*($U_{i},3$): The attacker *A* will get the smart card $SC_{i}$ held by the user $U_{i}$ through this corrupt inquiry and recover all stored information in the smart card through reverse engineering.

If attacker *A* makes three kinds of corrupt queries to the user $U_{i}$, the user $U_{i}$ is said to be completely corrupted. In addition, if an attacker *A* makes queries (*Corrupt*($U_{i},2$) and *Corrupt*($U_{i},3$)) to the user $U_{i}$, the attacker *A* can recover the export order through an offline dictionary attack, so in this case we also call the user $U_{i}$ completely corrupted. We will explain this further in the next section.

*Test*$\left(\Pi_{P}^{x}\right)$: This instruction can only be used for user instance or sensor node instance. It does not describe the attacker’s real attack ability but is used to measure the semantic security of protocol session key. To answer this instruction, a uniform coin toss is needed. Assuming that the participant instance $\Pi_{P}^{x}$ has accepted the protocol and generated the session key, if the coin toss result is 1, the real session key of the instance is returned, and if the coin toss result is 0, a random number equal to the session key is returned. The attacker’s goal is to guess the result of a coin toss when simulation *Test* inquiry. If the attacker succeeds in guessing the result of the coin toss, *A* is regarded as successful, and we record this event *Succ*.

In the above attack games, we need to define session freshness to exclude the situation where an attacker *A* can easily win the attack game. We limit that the attacker can implement *Test* inquiry to only new session instances. Defining user instances or sensor node instances is new if (1) Participants or their partners are not completely corrupted before the instance runs the protocol; (2) Attackers have not implement *Reveal* inquiry to the instance or its partner instances (if they exist).

Given a multifactor protocol *P*, the advantage of an attacker *A* to destroy the session key security of the protocol is defined as $Adv_{P.D}^{mfake}(A)=2.Pr[Succ]-1$. If for an attacker *A* with arbitrary probabilistic polynomial time, the advantage $Adv_{P.D}^{mfake}(A)$ of destroying the session key security of the protocol *P* is negligible, it is said that the multifactor protocol *P* satisfies the session key security.

### 5.2. Security Proof

**Theorem** **1.** *P is the multifactor protocol proposed in Section 4, and A is a probabilistic polynomial time attacker. Assuming that the symmetric encryption algorithm E used in the protocol is indistinguishably secure against selective message attacks h(•) is a random predictive function, and the fuzzy extractor used in P is robust, the advantage of the session key to attack the security of the multi-factor protocol is a negligible function concerning security parameters. That is to say*

$$Adv_{P.D}^{mfake}(A)\leq neg(l)$$

**Proof.**  We prove the security of the multifactor protocol in Section 4 by means of mixed game. We start with a real attack game and then gradually modify the simulated rules until the attacker has no advantage in differentiating session keys. For each attack experiment $Exp_{i}$, we use $Succ_{i}$ to represent the attacker’s attack advantage in this experiment; moreover, we use $\Delta_{i}$ to represent the difference between the experiment $Exp_{i}$ and the experiment $Exp_{i+1}$.

Exp0: This experiment simulates the attack game under the real protocol running conditions. From the definition of attacker advantage, we can know

$$Adv_{P.D}^{mfake}(A)=2Pr[Succ_{0}]-1$$

Exp1: In this experiment, we simulated random oracle function *h* by maintaining hash lists ^h. Specifically, for a random oracle function *h* query, assume that the input is *h*, the simulator first queries whether there is a record corresponding to *m* in the Hash list ^h and returns the corresponding output directly if it exists; otherwise, the simulator randomly selects a value from the range of the random oracle function as the output of the query and returns it to the attacker, and adds the corresponding record to the hash List ^h*. In addition, we use similar rules to simulate a private random oracle function $h^{*}$ and maintain the corresponding hash list ^h*. As can be seen from the above rules, the random oracle function is perfectly simulated, so we have

$$\Delta_{0}\leq neg(l)$$

Exp2: In this experiment, we modify the simulation rules of the passive conversation conducted by the attacker, that is, to modify the simulation of the *Execute* inquiry. Specifically, when an attacker implement *Execute* inquiry, all simulations are performed according to the real protocol description, but when calculating the session key $SK_{ij}$, we use the private random oracle function $h^{*}$ to calculate, and do not input the random number of users and sensor nodes, that is, we calculate $SK_{ij}=h^{*}(ID_{i}||ID_{SN_{j}}||T_{1}||T_{5})$. Correspondingly, when the user receives the last message, the input $SK_{ij}$, is calculated in the above way when validating the validity of $Auth_{1}$.

According to the randomness of random predictive function, experiment Exp2 and experiment Exp1 are indistinguishable unless the attacker implements $\left(ID_{i}||ID_{SN_{j}}||RN_{U_{i}}||RN_{SN_{j}}||T_{1}||T_{5}\right)$ inquiry to the random predictive function *h*. Because $RN_{U_{i}}$ is randomly chosen by users and transmitted by symmetric encryption algorithm in passive session, assuming that the attacker can get $RN_{U_{i}}$, the simulator can use the attacker’s decryption ability to attack the indistinguishable security of symmetric encryption algorithm. The simulator can use the challenge cipher-text of symmetric encryption algorithm as the cipher-text $C_{1}$ sent by users in the protocol. As the above-mentioned statute process is more intuitive, we will not elaborate on it for the sake of simplicity. From the above analysis, we can know:

$$\Delta_{1}\leq neg(l)$$

Exp3: In this experiment, we began to modify the simulation rules of active conversations with attackers, that is, to modify the simulation of *Send* inquiries. For queries *Execute*$Send(\Pi_{U_{i}}^{x},(Auth_{1},C_{3},T_{5}))$ received by user instances $\Pi_{U_{i}}^{x}$, if sensor nodes $SN_{j}$ are not corrupted by attackers, the simulator makes user instances $\Pi_{U_{i}}^{x}$ refuse to run the protocol without authentication. If the sensor node $SN_{j}$ is corrupted, the simulation is performed according to the protocol description, and the simulation rules are unchanged. Experiment Exp3 and experiment Exp2 are indistinguishable unless the attacker succeeds in obtaining the random number $RN_{U_{i}}$ chosen by the user and performs corresponding operations according to the protocol description to generate message $\left(Auth_{1},C_{3},T_{5}\right)$. Since sensor node $SN_{j}$ are not corrupted by attackers, attackers can only obtain random number $RN_{U_{i}}$’s information through encrypted cipher text. Similar to the analysis of the previous experiment, if an attacker can get information about random numbers, we can use the attacker’s decryption ability to attack the indistinguishable security of symmetric encryption algorithm. So, we have:

$$\Delta_{2}\leq neg(l)$$

Exp4: In this experiment, we continue to modify the simulation rules for active conversations with attackers. For the $Send(\Pi_{SN_{j}}^{z},(ID_{MN_{j}},C_{2}))$ queries received by the sensor node instance $\Pi_{SN_{j}}^{z}$, if the message $C_{2}$ is not generated by the gateway in the corresponding session, we make the sensor node instance $\Pi_{SN_{j}}^{y}$ reject and terminate the protocol operation directly. Because sensor nodes $SN_{j}$ are not corrupted by attackers, attackers cannot use their master key $MK_{SN_{j}}$ to encrypt a fresh timestamp. Otherwise, we can choose two identical messages like $\left(ID_{i},ID_{SN_{j}},RN_{U_{i}},T_{1},*\right)$ inquiry in the attack game against symmetric encryption algorithm. The last one of the messages chooses the current timestamp of the system and the other one chooses the previous timestamp. The simulator chooses one of them. Encryption as a challenge cipher text. In this way, the indistinguishable security of symmetric encryption algorithm for selective message attacks can be destroyed by attackers’ attacks on protocols. So, we have:

$$\Delta_{3}\leq neg(l)$$

Exp5: In this experiment, we continue to modify the simulation rules for active conversations with attackers. When the gateway instance $\Pi_{GW}^{y}$ receives $Send(\Pi_{GW}^{y},(DID_{i},ID_{SN_{j}},C_{1},T_{1}))$ from the attacker, it first queries the identity $ID_{i}$ of the real user which the attacker counterfeit with $DID_{i}$. If the user has been corrupted completely by the attacker, then the simulation rules are carried out according to the description of the protocol without any change. If the user’s password and biological template are corrupted by the attacker, the simulator makes the gateway instance refuse directly and terminate the protocol operation. Experiment Exp5 and experiment Exp4 are indistinguishable unless the attacker can recover $h(ID_{i}||DID_{i}||X_{S})$ without getting the information in the smart card. For the high entropy of $X_{S}$ and the randomness of the random predictive function, the probability of the attacker’s recovery $h(ID_{i}||DID_{i}||X_{S})$ without the data in the smart card is negligible. So, we have:

$$\Delta_{4}\leq neg(l)$$

Exp6: In this experiment, we last modified the simulation of an attacker’s active conversation. Similar to the experiment Exp5, when the gateway instance $\Pi_{GW}^{y}$ receives the message $Send(\Pi_{GW}^{y},(DID_{i},ID_{SN_{j}},C_{1},T_{1}))$ from the attacker, it first queries the identity $ID_{i}$ of the real user which the attacker counterfeit with $DID_{i}$. If the user has been completely corrupted by the attacker, then the simulation rules are carried out according to the description of the protocol without any change. If the user’s password and smart card are corrupted by the attacker, the simulator makes the gateway instance refuse directly and terminate the protocol operation. Experiment Exp6, and experiment Exp5 are indistinguishable unless an attacker can recover $h(ID_{i}||DID_{i}||X_{S})$ without an effective biological template. Because what is stored in the smart card is $h(ID_{i}||DID_{i}||X_{S})⊕RPW_{i}$, the attacker must restore the biological key $\sigma_{i}$ to calculate $RPW_{i}$, and then the attacker can restore $h(ID_{i}||DID_{i}||X_{S})$. From the security of the fuzzy extractor, it can be seen that under the condition of only public information $\tau_{i}$, the uniform distribution of the biological key $\sigma_{i}$ and the range of the fuzzy extractor is statistically indistinguishable. So, we have:

$$\Delta_{5}\leq neg(l)$$

In the above experiments, the session keys in all passive sessions are randomly selected after constant modification of protocol simulation rules. In all active attack sessions conducted by an attacker, if the attacker’s counterfeited participants are not completely corrupted, the active session will be rejected according to simulation rules (when the attacker’s counterfeited participants are completely corrupted, the session is not new). Fresh, so the security of session key cannot be guaranteed. Therefore, in Exp5, the attacker’s advantage in distinguishing session key from random number is 0.

Combining the conclusions of all the above mixed experiments, Theorem 1 is proved. □

Note: When defining a user’s complete corruption in the security model, we define that the user is also completely corrupted when the attacker gets the user’s biological template and smart card. Correspondingly, in the proof of Theorem 1, we do not consider the counterfeit attack when the attacker gets the biological template and smart card, because the attacker can guess the password offline and verify the password guess by the message $\left(DID_{i},ID_{SN_{j}},C_{1},T_{1}\right)$. In the above attack scenario, the attacker can recover the user password through offline dictionary attack. As pointed out in document [22], any multifactor protocol without public key cryptosystem cannot resist the attack mentioned above. The above-mentioned problem is still an open and difficult one. In our improved protocol, we bind the user’s real identity and password. Attackers need to guess the identity information and password at the same time. Usually, the identity information and password are 32 bits, respectively. Therefore, the ability of the protocol to resist dictionary attacks is enhanced to a certain extent.

## 6. Performance Analysis

In this section, we compare the computational cost, communication costs and the functionality features of the improved protocol with those of other similar protocols [3,6,8,10,18]. Since each user registers once, we focus on the login, authentication & key establishment phases for comparison of computational/communication cost. We are using $T_{H}$, $T_{sym}$, $T_{f}$, $T_{epm}$ and $T_{pub}$ to denote the time complexity of the output of a hash operation, a symmetric encryption/decryption operation, a fuzzy extractor operation, an elliptic curve point multiplication operation, and a public key encryption/decryption operation respectively. The time complexity of a fuzzy extractor operation is higher than the time complexity of a hash operation. Comparison of computational costs is shown in Table 2.

For communication cost, we compare the number of rounds and bandwidth. We assume that a random number, a point on an elliptic curve group, and an output of hash function be 160 bits long; the identity and the password be 32 bits long; and the timestamp be 64 bits long. The cipher-text length of the symmetric encryption algorithm is the same as that of the plain-text, while the cipher-text length of the public key encryption algorithm is set twice that of the plain-text.

From Table 2, we can see that the computational cost of our improved protocol is comparable to that of the protocol in [18], higher than that in [3,6,8], but significantly better than that of the protocols in [10,12]. However, the protocol in [3,6] only achieves authentication and does not establish session keys for users and sensor nodes. The computational cost of the protocol in [3,6] will be comparable to our improved protocol after increasing computational complexity to achieve key generation.

As can be seen from Table 3, the number of communication rounds of our improved protocol has reached the optimum level, which is slightly higher in bandwidth than that of the protocols in [3,6,8]. The protocols in [3,6] save some communication bandwidth because it does not establish session key between the user and the sensor. However, the protocol in [8] uses the "challenge-response" mechanism, which leads to the high number of communication rounds. In wireless networks, reducing the number of communication rounds is far more important than reducing the computational cost and communication bandwidth and this feature is achieved by the proposed protocol.

Next, we focus on the communication bandwidth on sensor-node (SN) as apparent from Table 3. For this specific comparison, we have considered incoming as well as outgoing messages on SN because when a SN receives any message it also exhausts its memory as well as battery power. We observe from Table 3 that the total communication bandwidth on sensor-node (SN), is least in the protocols in [3,6], highest in the protocol in [18], and it is same for our improved protocol & the protocol in [12]. However, if we consider the communication bandwidth in the context of the messages communicated by SN then, it is nil in the protocols in [3,6]; it is least in the protocol in [10]; and it is highest in the protocol in [12].

Besides, the average communication costs between the user and a sensor is given by 384 bits and 448 bits communication bandwidth in the protocol in [18] and the proposed protocol respectively; and it is nil in the remaining protocols [3,6,8,10] because the protocols in [3,6,8,10] do not allow the user to access the real time data directly from the SN.

Table 4 compares the functionality features of the proposed protocol with the protocols in [3,6,8,10,18]. The protocol in [10] is said to provide only partial three-factor security because it uses simple hash function for handling the biometrics of the user which does not offer correct biometrics-matching. Further, it is noticeable that the protocol in [10] provides formal security analysis using BAN-Logic instead of using random oracle model or the standard model.

The protocols in [8,10] are said to provide only partial mutual authentication because these protocols do not allow a user to verify the legitimacy of the SN. Out of all the protocols considered for comparison, only the protocols in [12,18] and our improved protocol provides access of real-time-data to the user from sensor node. In the sensitive applications of WSNs, the direct access of the real time from SN is crucial for decision making. Further, the protocol in [18] does not resist node capture attack and denial-of-service attack. Table 4 shows that the protocol in [12] and our improved protocol satisfy the maximum number of functionality features. But our improved protocol is more suitable for applications in WSNs owing to its low cost.

According to the above discussion, our improved protocol offers high functionality without adding much at the computational/communication cost, so it is more suitable for the application requirements of WSNs.

## 7. Conclusions

Security and privacy issues are the most concerns in various IoT applications and environments [23,24,25,26,27,28,29,30]. This paper analyses the security of a multi-factor authentication protocol for WSNs with the provision of privacy protection. This paper points out that the Das’s protocol cannot resist node capture attack, denial of service attack, and does not realize the security of a real multi-factor protocol. Therefore, we have improved the security vulnerabilities of the Das’s protocol by proposing an improved protocol. We have formally proved the security of the proposed protocol in random oracle model. We have justified the efficiency and security of the proposed protocol by comparing it with the recent and related protocols. We have realized that at present, the design of multi-factor protocol in wireless sensor networks is not standardized; especially the research on security model is not sufficient. The results of this paper once again verify the importance of security proof for authentication protocols. In future work, we will systematically summarize the security requirements of multi-factor protocols in wireless sensor networks, improve the security model of multi-factor protocols in wireless sensor networks, and will try to design more secure and efficient multi-factor protocols under the guidance of our improved model.

## Figures and Tables

**Table 1:** The symbols and definitions used in this paper.

Symbol	Definition
$U_{i}$	The *i* th user
$ID_{i}$	The identity information of user $U_{i}$
$PW_{i}$	The password of user $U_{i}$
$B_{i}$	The biological sample of user $U_{i}$
K	The high entropy key of user $U_{i}$
GW	The gateway node
$SN_{j}$	The *j* th sensor node in the WSN
$ID_{SN_{j}}$	Identity information of the *j* th sensor node
$MK_{SN_{j}}$	The master key of the *j* th sensor node
h(·)	Anti-collision cryptographic one-way hash function
$X_{S}$	Master key of the gateway node *GW*
$E_{k}(m)$	Encrypt the plain-text *m* with a key *k* using an encryption algorithm
$D_{k}(m)$	Using a decryption algorithm to decrypt a cipher text *m* using a key *k*
$RN_{X}$	Participant *X* generated random number
$T_{i}$	Current timestamp of the system
ΔT	Maximum transmission delay allowed in within WSNs
⊕	Bit XOR operation
||	Data cascading operation

**Table 2:** Comparison of computational cost.

Compared Protocols	Users	Gateway Nodes	Sensor Nodes
Reference [3]	$4T_{H}$	$4T_{H}$	$T_{H}$
Reference [6]	$5T_{H}$	$5T_{H}$	$T_{H}$
Reference [8]	$2T_{H}$	$5T_{H}$	$2T_{H}$
Reference [10]	8$T_{H}$+$T_{pub}$	8$T_{H}$+$T_{pub}$	$2T_{H}$
Reference [12]	$T_{f}$+ 11$T_{H}$+2$T_{epm}$	10$T_{H}$	3$T_{H}$+2$T_{epm}$
Reference [18]	$T_{f}$+ $7T_{H}$	2$T_{H}$+$T_{sym}$	2$T_{H}$+$T_{sym}$
Protocol in this paper	$T_{f}$+ 5$T_{H}$+2$T_{sym}$	2$T_{H}$+2$T_{sym}$	3$T_{H}$+2$T_{sym}$

**Table 3:** Comparison of communication cost.

Compared Protocols	Rounds of Communication	Overall Bandwidth of Communication	Bandwidth of Communication on SN
Reference [3]	3	832 bits	224 bits (GW to SN)
Reference [6]	3	928 bits	224 bits (GW to SN)
Reference [8]	8	1056 bits	352 bits (SN to GW) 160 bits (GW to SN) Total = 512 bits
Reference [10]	4	1600 bits	224 bits (SN to GW) 256 bits (GW to SN) Total = 480 bits
Reference [12]	4	2336 bits	480 bits (SN to GW) 352 bits (GW to SN) Total = 832 bits
Reference [18]	3	1376 bits	384 bits (SN to U) 544 bits (GW to SN) Total = 928 bits
Our improved protocol	3	1216 bits	448 bits (SN to U) 384 bits (GW to SN) Total = 832 bits

**Table 4:** Comparison of functionality features.

	Protocol						
Functionality	[3]	[6]	[8]	[10]	[12]	[18]	Ours
Provides password change facility	No	Yes	Yes	Yes	Yes	Yes	Yes
Provides mutual authentication	No	No	Partial Yes	Partial Yes	Yes	Yes	Yes
Provides three-factor security	No	No	No	Partial Yes	Yes	Yes	Yes
Resists node capture attack	No	No	No	No	Yes	No	Yes
Resists denial-of-service attack	No	No	No	No	Yes	No	Yes
Provides user anonymity	No	Yes	No	Yes	Yes	Yes	Yes
Provides key agreement	No	No	No	No	Yes	Yes	Yes
Provides formal security analysis	No	No	Yes	Yes	Yes	Yes	Yes
Provides access of real-time-data to U from SN	No	No	No	No	Yes	Yes	Yes
